# Supplementary figures and images for: E-Cadherin Is Expressed in Epithelial Cells of the Choroid Plexus in Human and Mouse Brains
Source: Curr Issues Mol Biol. 2023 Sep 26;45(10):7813–26. doi: 10.3390/cimb45100492 (PMC10605538; doi:10.3390/cimb45100492)

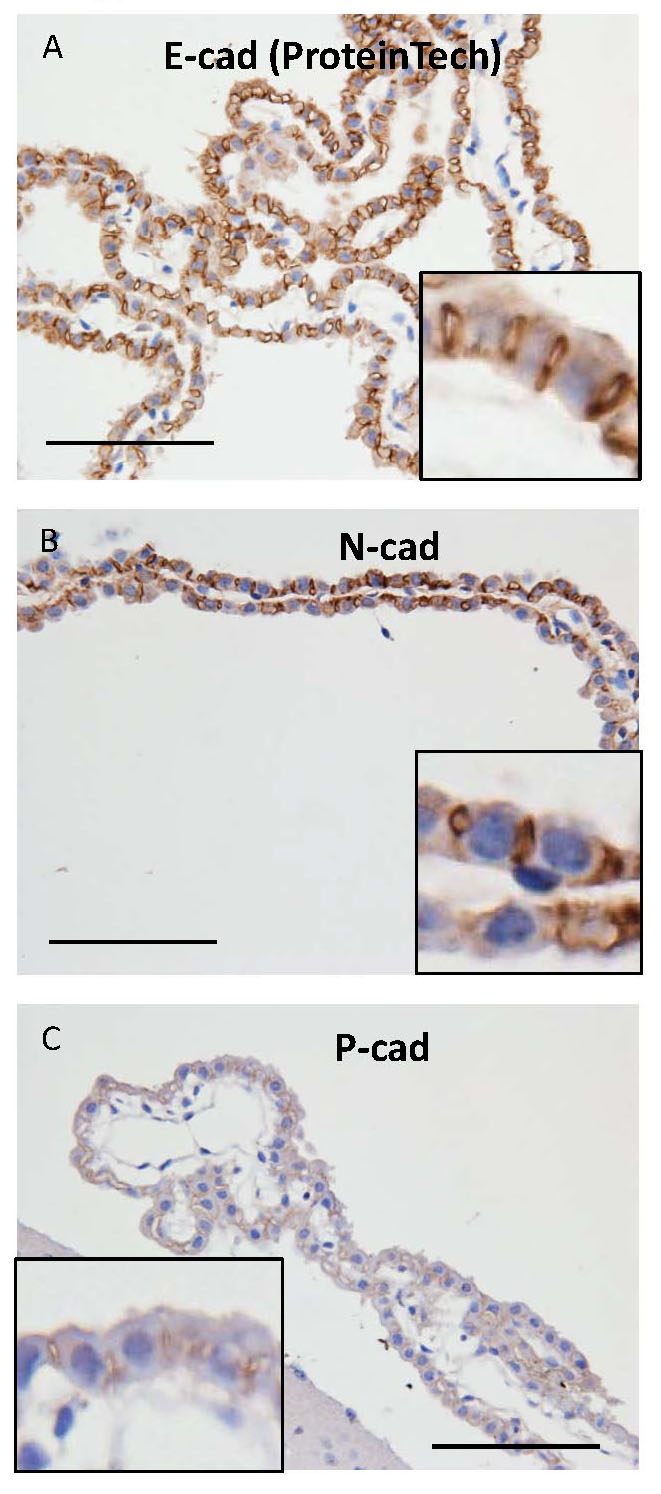

Supplement: Supplementary file 1 [file cimb-45-00492-s001.zip › 2023-CIMB-rev1-SFigs/2023-CIMB-SFig1-20230828.jpg]

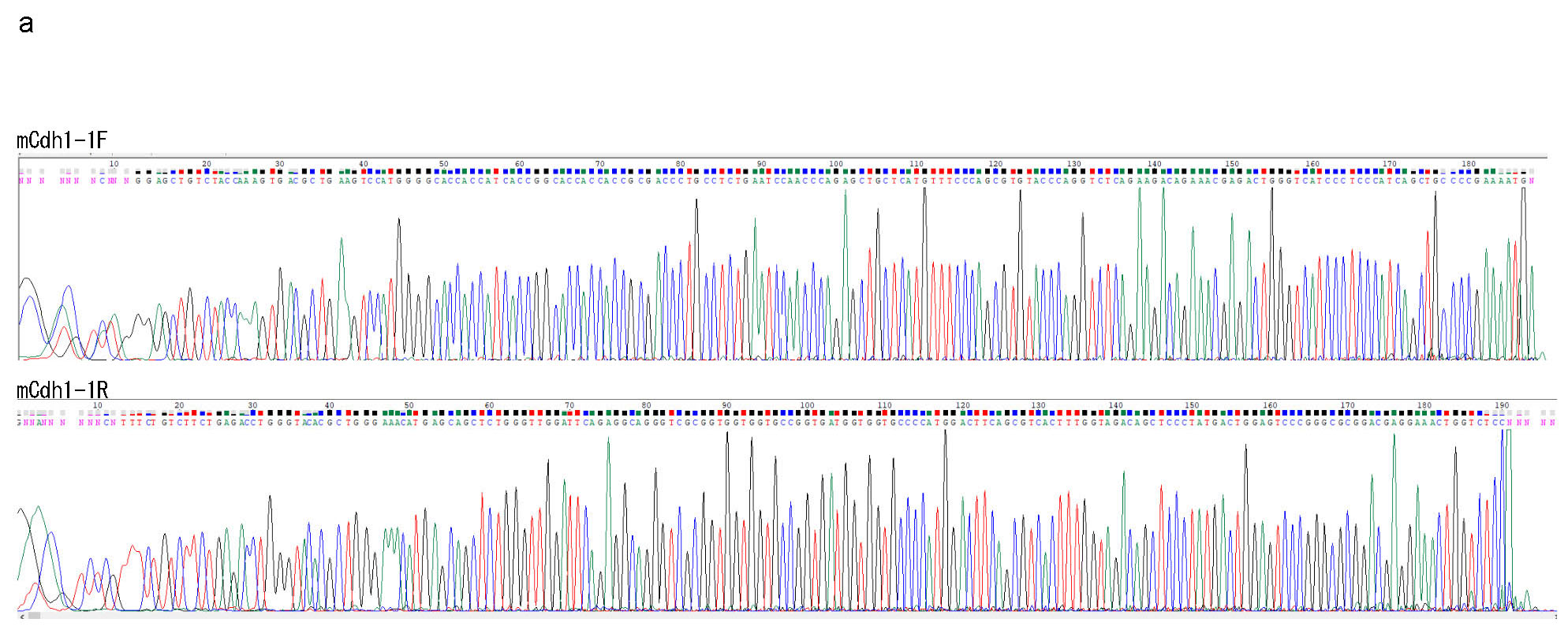

Supplement: Supplementary file 1 [file cimb-45-00492-s001.zip › 2023-CIMB-rev1-SFigs/2023-CIMB-SFig2a.jpg]

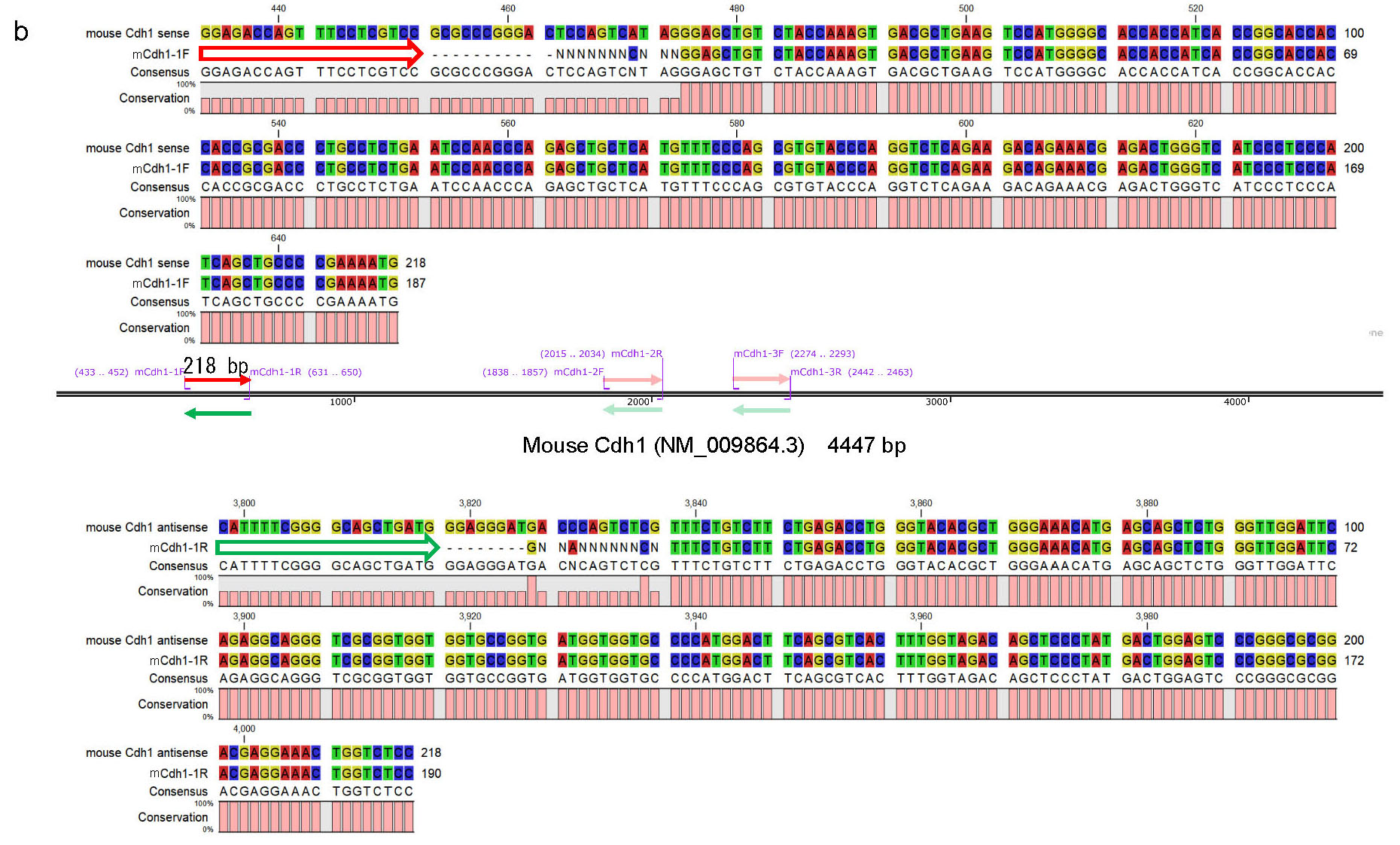

Supplement: Supplementary file 1 [file cimb-45-00492-s001.zip › 2023-CIMB-rev1-SFigs/2023-CIMB-SFig2b.jpg]

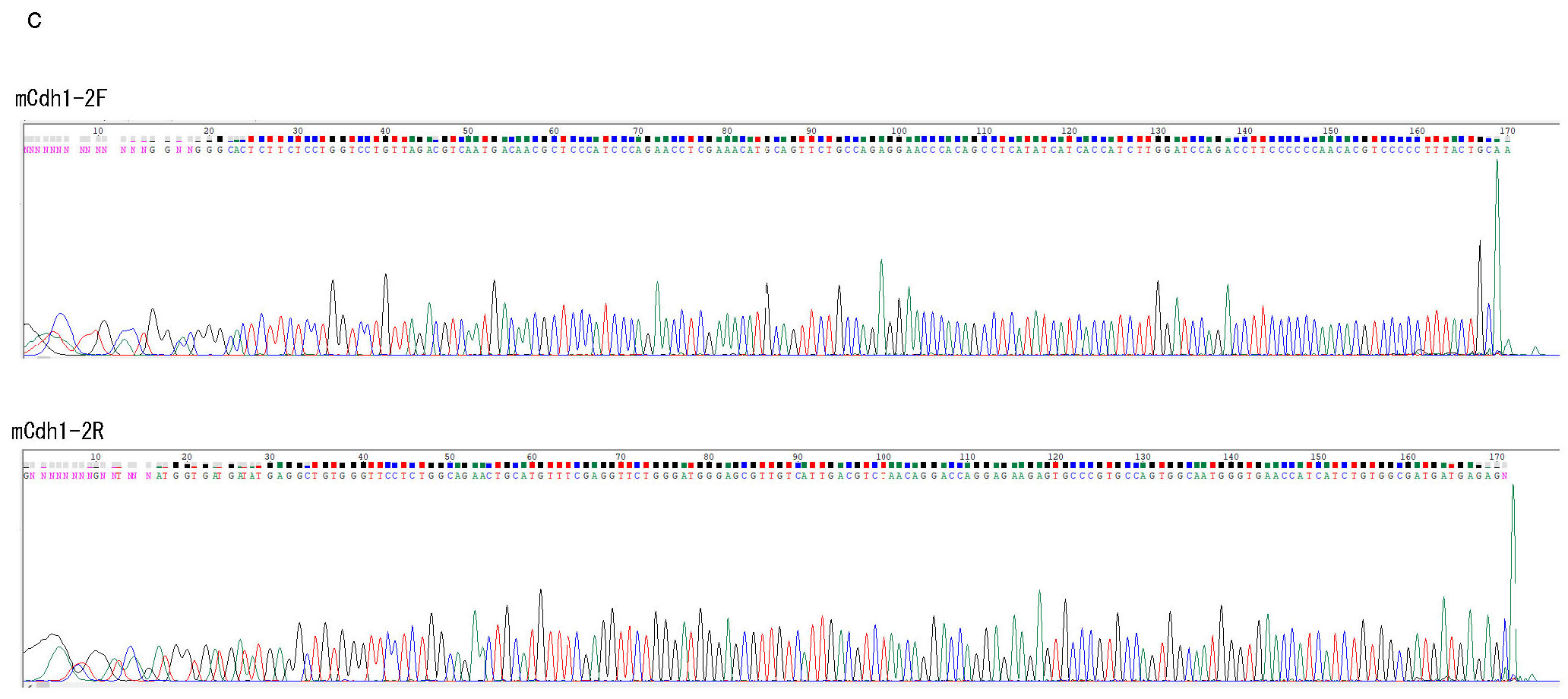

Supplement: Supplementary file 1 [file cimb-45-00492-s001.zip › 2023-CIMB-rev1-SFigs/2023-CIMB-SFig2c.jpg]

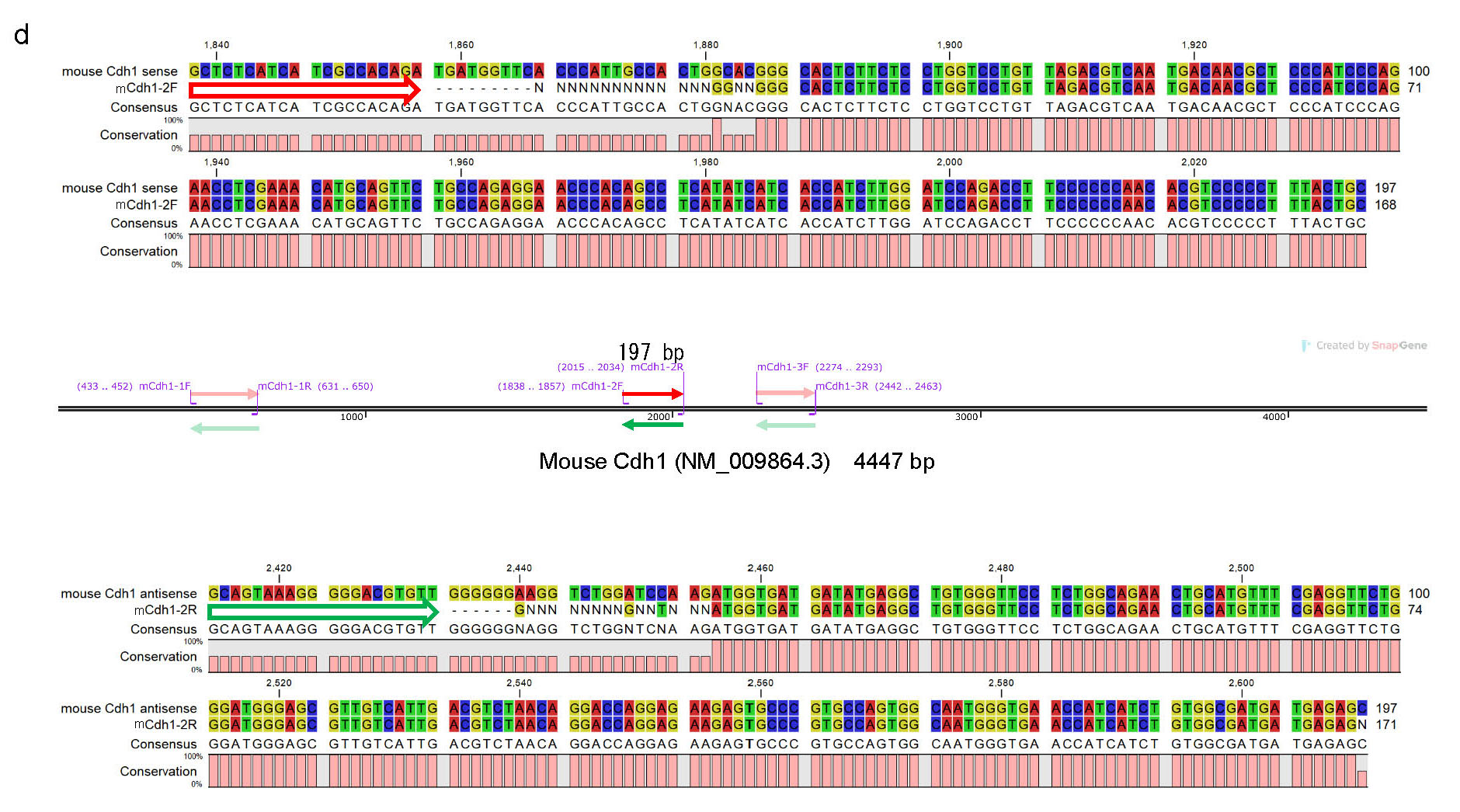

Supplement: Supplementary file 1 [file cimb-45-00492-s001.zip › 2023-CIMB-rev1-SFigs/2023-CIMB-SFig2d.jpg]

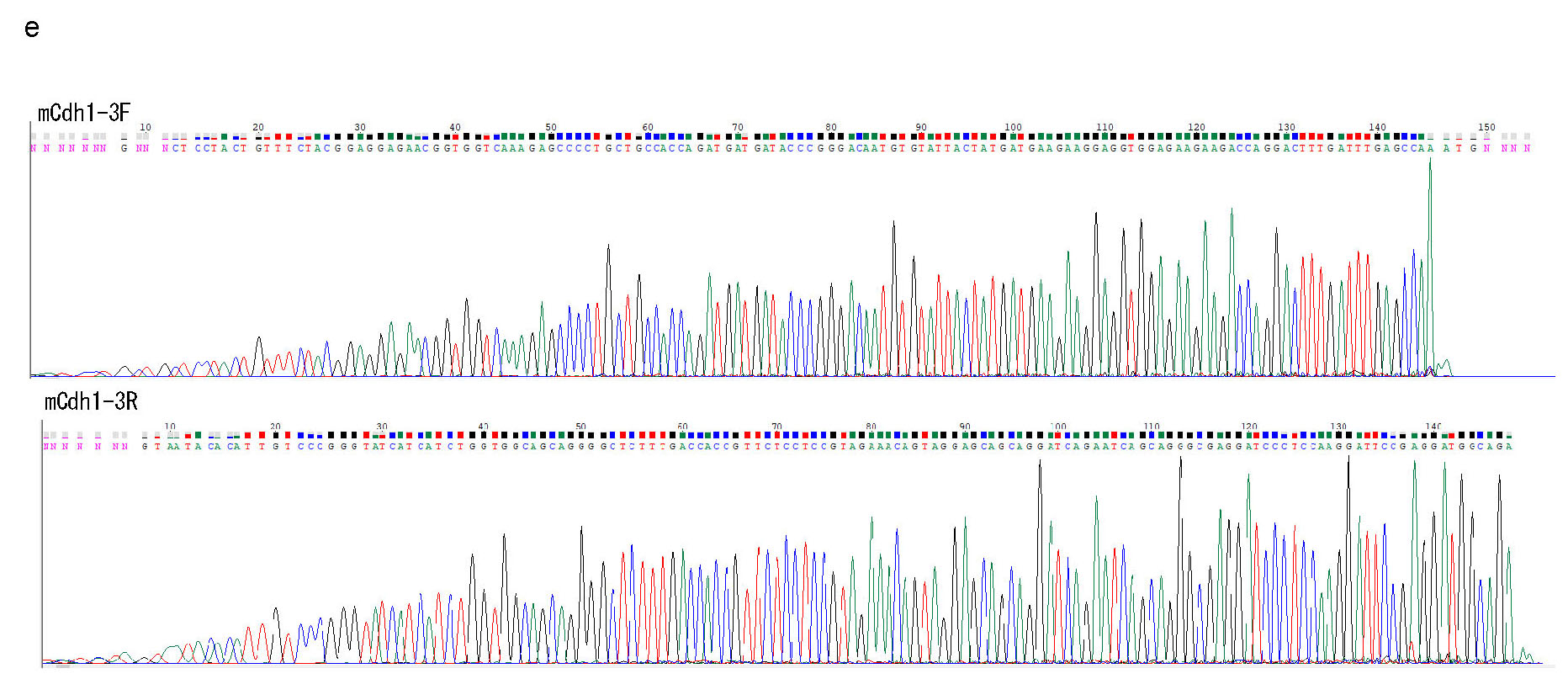

Supplement: Supplementary file 1 [file cimb-45-00492-s001.zip › 2023-CIMB-rev1-SFigs/2023-CIMB-SFig2e.jpg]

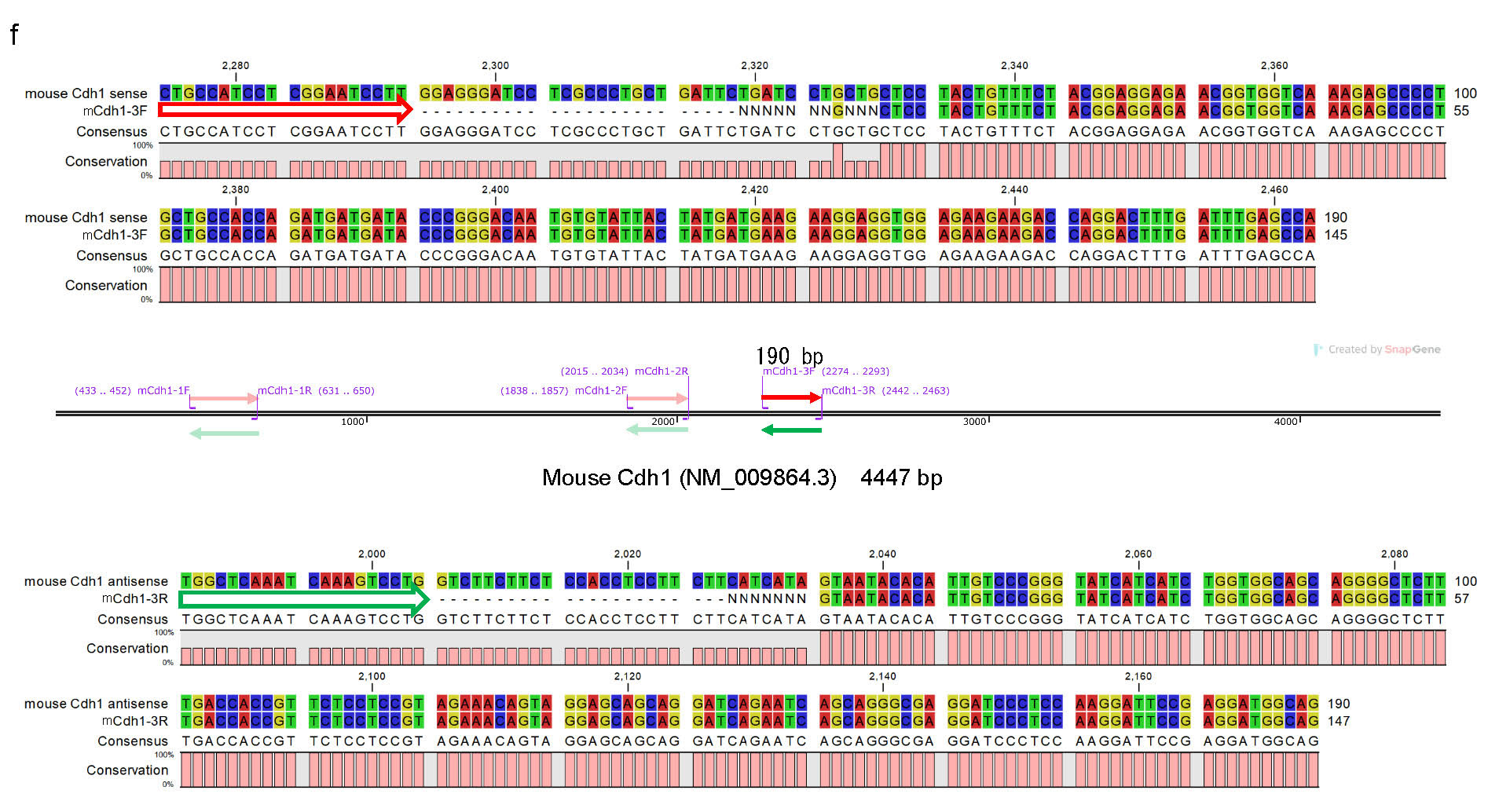

Supplement: Supplementary file 1 [file cimb-45-00492-s001.zip › 2023-CIMB-rev1-SFigs/2023-CIMB-SFig2f.jpg]

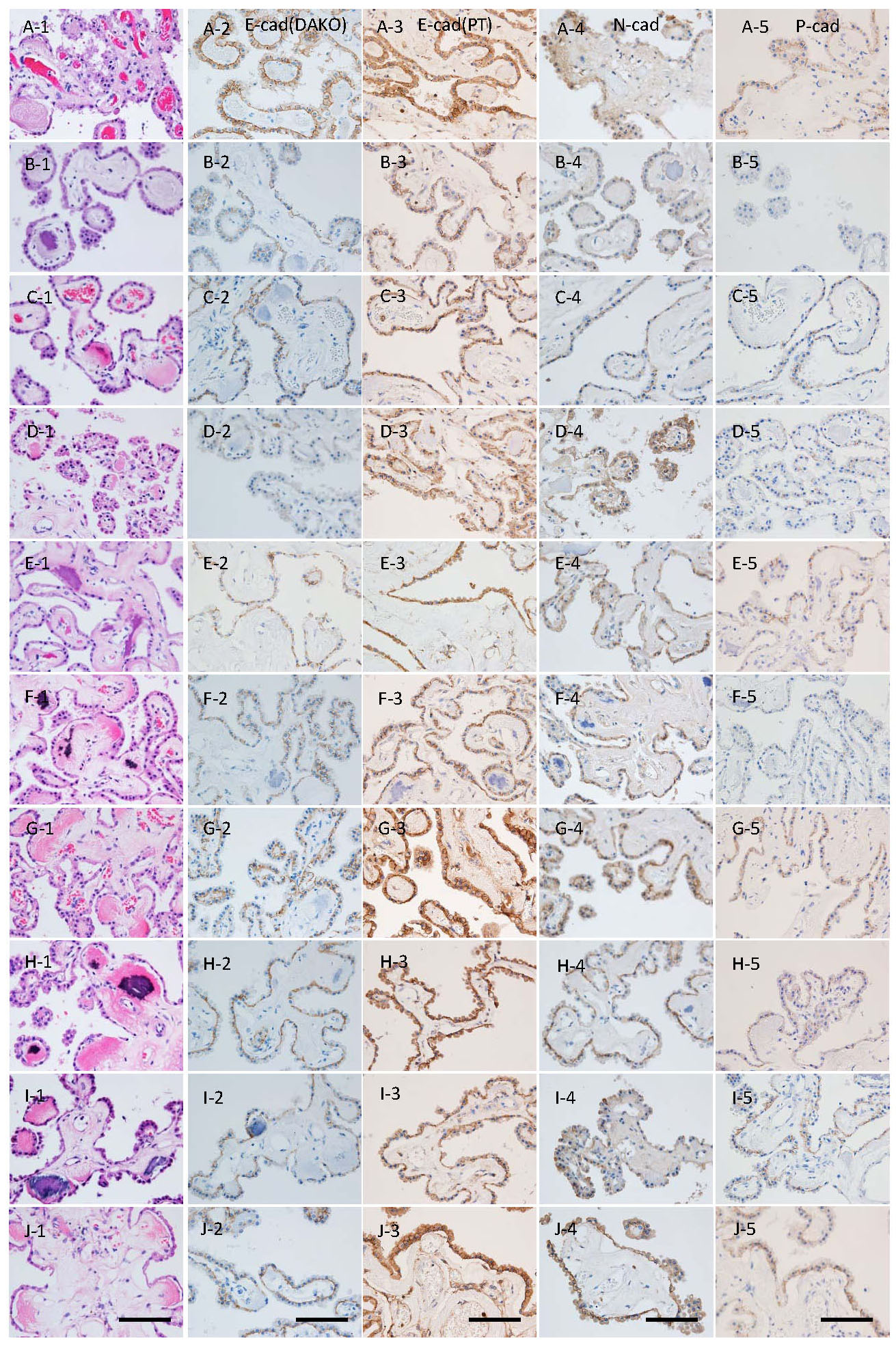

Supplement: Supplementary file 1 [file cimb-45-00492-s001.zip › 2023-CIMB-rev1-SFigs/2023-CIMB-SFig3-20230916.jpg]
